# COVID-19’s Impact on Health Professionals’ Quality of Professional Life: A Single-Site Cross-Sectional Study

**DOI:** 10.3390/healthcare14020279

**Published:** 2026-01-22

**Authors:** Michael Rovithis, Sofia Koukouli, Anastasia Konstantinou, Maria Moudatsou, Nikos Rikos, Manolis Linardakis, Konstantinos Piliotis, Areti Stavropoulou

**Affiliations:** 1 Department of Business Administration & Tourism, School of Management and Economics Sciences, Hellenic Mediterranean University, 71410 Heraklion, Greece; 2 Department of Social Work, School of Health Sciences, Hellenic Mediterranean University, 71410 Heraklion, Greece; koukouli@hmu.gr (S.K.); moudatsoum@hmu.gr (M.M.); 3 Venizeleio & Pananio General Hospital, 71409 Heraklion, Greece; natasako@hotmail.com; 4 Department of Nursing, School of Health Sciences, Hellenic Mediterranean University, 71410 Heraklion, Greece; rikosn@hmu.gr; 5 Department of Social Medicine, School of Medicine, University of Crete, 70013 Heraklion, Greece; linman@med.uoc.gr; 6 University General Hospital of Crete, 71500 Heraklion, Greece; pilio68@yahoo.gr; 7 Department of Nursing, Faculty of Health Science, West Attica University, 12243 Athens, Greece; astavropoulou@uniwa.gr

**Keywords:** quality of professional life, compassion fatigue, burnout, compassion satisfaction, secondary post-traumatic stress, COVID-19

## Abstract

Background/Objectives: Professional quality of life influences patient care, staff well-being, and organizational efficacy. The COVID-19 pandemic placed pressure on healthcare professionals, disrupting their professional quality of life and imposing a psychological burden. In Greece, these issues were intensified by a decade of economic crisis, marked by constrained healthcare budget, personnel shortages, and insufficient resources. This study investigates the pandemic’s impact on the professional quality of life of Greek healthcare professionals to support targeted interventions. Methods: A cross-sectional study was conducted using descriptive statistics. The participants were a convenience sample of 246 healthcare professionals from a Greek regional university hospital with at least one year of experience and who had worked with COVID-19-positive or potentially exposed but asymptomatic patients. Data were collected between March and June 2021 using the Professional Quality of Life Scale (version 5). Results: Of the 246 participants, 81.3% were women and 33.8% were aged 50 or older. Moderate concern and fear regarding COVID-19 were reported, with 34.6% extremely afraid of transmitting the virus to family or friends and 22.8% to patients or their families. Overall professional quality of life was moderate: compassion satisfaction was moderate to high, while burnout and secondary traumatic stress were moderate to low. Higher compassion satisfaction was linked to holding a position of responsibility. Burnout was associated with having children, permanent employment, years of experience, and increased pandemic-related fear. Higher secondary traumatic stress was associated with older age, more years of experience, and greater pandemic-related fear. Conclusions: These findings support international research and highlight that the moderate levels observed indicate intrinsic motivation based on professionalism in patient care, providing evidence of resilience and coping mechanisms that reduce psychological consequences on well-being due to the pandemic.

## 1. Introduction

Professional quality of life (ProQol) is an essential component of overall quality of life, as it indicates the extent to which individuals experience well-being in their professional roles. The concept of ProQol is often considered synonymous with employee well-being [1], yet it remains distinct from job satisfaction [2]. ProQol encompasses the broader context of an employee’s work environment and overall occupational experience—including job satisfaction, work–life balance, safety, fair compensation, and opportunities for career growth. Conversely, job satisfaction refers primarily to the emotional response and sense of accomplishment that a person receives from their employment [3]. The fundamental components of ProQol include job satisfaction, social relations in the workplace, stress, overall life quality, safety, societal status and salary, work–life balance, legitimacy, and opportunities for growth [4].

Multiple definitions of ProQol have been proposed in the literature worldwide. Recent perspectives highlight its multidimensional and subjective nature, emphasizing its connection to both personal and professional aspects of life [5]. Stamm defined ProQol as the quality experienced by individuals engaged in professions that serve the community [6]. This construct is inherently complex, as it relies on both work environment characteristics (e.g., organizational features and job tasks) and the personal traits of employees. The author recognized two primary dimensions of ProQol: a positive dimension—compassion satisfaction (CS)—and a negative dimension—compassion fatigue (CF). CS is the positive emotion that a person feels while helping or caring for someone else or other living things. CF is the cost of caregiving, characterized as the stress response prevalent among healthcare professionals (HPs) [7]. CF is related to both burnout (BO) and secondary traumatic stress (STS). BO, characterized by fatigue, frustration, anger, and despair typically associated with one’s occupation, constitutes the primary aspect, while STS is linked to exposure to stressful events resulting in fear, sleeping problems, intrusive thoughts, or avoidance [8]. The present study adopts Stamm’s dual-dimensional theoretical framework to assess and investigate ProQol.

Accordingly, job satisfaction is an important element of ProQol, defined by the existence of positive and pleasant attitudes and emotions among employees regarding their work environment [9]. While high job satisfaction has a positive effect on the performance of employees, decreased job satisfaction may lead to burnout, absenteeism, and even leaving the profession. Job satisfaction among HPs is crucial to any organization’s quality and productivity [10].

Current research findings demonstrate a significant association between job satisfaction and ProQol among HPs, wherein higher levels of CS—the positive aspect of ProQol—correlate with increased job satisfaction, whereas BO and STS are associated with decreased satisfaction levels. A cross-sectional study of nurses in tertiary hospitals in Ethiopia revealed a strong correlation between CS and job satisfaction, highlighting their interrelation in clinical practice and well-being results [11].

The World Health Organization (WHO) characterizes an attractive and supportive workplace as one that draws individuals into health professions, promotes retention, and enhances effective performance [12].

The outbreak of the COVID-19 pandemic significantly disrupted the quality of life of HPs, affecting their physical, psychological, and social well-being [13]. Under extreme pressure, HPs were required to make ethically complex decisions to save patients’ lives, such as allocating limited resources fairly, balancing personal well-being with professional responsibilities, and maintaining medical care despite insufficient resources [13,14]. While the general population reduced social interactions and activities to minimize infection risk, HPs encountered increasing workloads, prolonged shifts, limited resources, and in some cases, hazardous infrastructure. The use of personal protective equipment (PPE) caused physical discomfort and breathing difficulties [15]. Additionally, fear of infection and concerns about potential transmission of the virus to family, friends, or colleagues [16] contributed to social isolation and restricted social networks [17]. Fear of infection even led many HPs to seriously consider resignation or withdrawal from their duties [18]. The WHO estimated that between 80,000 and 180,000 healthcare and care workers may have died from COVID-19 between January 2020 and May 2021, with a mean estimate of 115,500 fatalities [19]. During the pandemic, one in four HPs reported depression or anxiety, and one in three experienced insomnia [20]. Bozdağ and Ergün confirmed the physical and psychological exhaustion of healthcare staff, identifying increased responsibilities, demanding schedules, and exposure to infected patients as key contributing factors [21]. Furthermore, the global variability in the acceptance of the COVID-19 vaccine, not only among the general population but also among HPs, contributed to an additional workload and exacerbated their psychological distress [22].

The pandemic imposed an enormous strain on HPs, increasing workloads and disrupting work–life balance, which led to increased stress, burnout, and a decline in both quality of life and patient care, especially in compassionate care delivery [23,24,25]. For the first time, HPs had to deal with conflicting thoughts and challenges in balancing professional roles and family responsibilities. Work-related concerns, along with psychological strain, moral dilemmas, and patient expectations, further burdened their emotional state and significantly reduced their professional quality of life [26]. Nevertheless, it is essential to acknowledge that ProQol for HPs considerably differs based on factors such as country or region, urban or rural environments, private or public facilities, and the unique challenges present within each healthcare system [27].

At the onset of the pandemic, Greece faced a health crisis while still struggling with the consequences of the financial crisis. The healthcare system was severely fragmented, operating within an outdated organizational framework and plagued by numerous inefficiencies, including excessive centralization in decision-making and administrative processes, inadequate planning and coordination, limited managerial and administrative capacity, and the inequitable and inefficient distribution of human and economic resources, low staffing levels, and a significant burden on HPs [28,29].

The overall findings of these studies clearly demonstrate the multidimensional physical, psychological, and social consequences encountered by HPs during the COVID-19 pandemic. These results directly and significantly affected their ProQol, emphasizing the need for a systematic and comprehensive investigation of this phenomenon.

This study investigates the pandemic’s impact on the ProQol of HPs at a University General Hospital in Greece during the COVID-19 pandemic. Specifically, the research aims to assess the ProQοL levels among HPs and examine the extent to which these levels are associated with pandemic-related concerns, with a particular emphasis on the fear of viral transmission. Furthermore, the study explores whether specific demographic and occupational characteristics serve as significant predictors or correlates of the participants’ ProQol.

An in-depth exploration of the pandemic’s impact on the ProQol of HPs in a Greek hospital will add valuable insights to the existing scientific knowledge, thereby enhancing the understanding of HPs’ work well-being and the consequences of the pandemic. This understanding will facilitate the development of targeted interventions to mitigate or prevent similar adverse outcomes in future pandemics or health crises.

## 2. Materials and Methods

### 2.1. Study Design

This single-site cross-sectional study aimed to investigate the ProQol of life of HPs at a University General Hospital in Greece during the COVID-19 pandemic.

### 2.2. Study Population-Survey Instrument

The study population consisted of HPs with at least one year of experience and who had worked with COVID-19-positive or potentially exposed but asymptomatic patients working at a university general hospital in a regional area of Greece at the time of the study. This specific hospital, with a total capacity of 676 beds, was selected for its essential and multifaceted role, as it was designated as the main tertiary referral center during the COVID-19 pandemic, not only for Crete but for southern Greece as a whole. Data was collected through paper-based questionnaires from March to June 2021 using a convenience sampling method. In accordance with the rigorous pandemic restrictions and the operational guidelines of the hospital, an invitation to participate was disseminated, resulting in the involvement of healthcare professionals from eight hospital sectors: pathology clinics (*n* = 113), surgery (*n* = 67), laboratory (*n* = 26), mental health (*n* = 14), units (*n* = 6), and other departments (*n* = 20). The Professional Quality of Life Scale, 5th Edition (ProQol-v5) [6], was utilized to assess HPs’ ProQol. The instrument comprises 30 closed-ended items assessing various aspects of ProQol. Responses are rated on a five-point Likert scale ranging from “never” to “very often.” The scale measures three core dimensions: compassion satisfaction (CS), secondary traumatic stress (STS), and burnout (BO). The second section of the questionnaire included eight items to assess specific contamination-and-transmission fears related to the COVID-19 pandemic. The five Likert-type items were not intended to constitute a previously validated standardized scale, but rather an ad hoc, context-specific measure designed to capture healthcare professionals’ fear of COVID-19 infection and transmission during the pandemic. Items were created based on a relevant article about transmission fear concerning coronavirus [30]. Interviews with HPs regarding their concerns and fears related to COVID-19 also aided the development of the items. The first three dichotomous questions investigated HPs’ contact at work with COVID-19 positive patients with or without hospitalization, as well as with asymptomatic patients who may have been exposed to the coronavirus. The next five items evaluated HPs’ fear concerning the risk of infection or virus transmission. Participants’ replies were documented via a five-point Likert scale, with values ranging from 1 (“Not at all”) to 5 (“Very much”). The third section consisted of closed-ended demographic questions, including gender, age, marital status, educational level, and years of professional experience, among other sociodemographic characteristics.

### 2.3. Ethics

Prior to study initiation, ethical approval was obtained from the Scientific Board of the 7th Health Region of Crete (Ref. No. 25051/16/06/2021). All procedures were conducted in accordance with the ethical standards of the institutional research committee and the principles outlined in the Declaration of Helsinki (revised 2013) [31]. Each participant received detailed written and verbal information about the study’s purpose, procedures, potential risks, and anticipated benefits. Written informed consent was obtained from all participants prior to data collection. Voluntary participation was emphasized, and participants were informed of their right to withdraw from the study at any time without any penalty or consequence.

### 2.4. Statistical Analysis

Data analysis was performed using the IBM SPSS Statistics for Windows, Version 26.0 (IBM Corp., Armonk, NY, USA). Descriptive statistics were used to calculate frequency distributions of the participants’ demographic and occupational characteristics. Differences in response distributions on the fear of coronavirus transmission and the Professional Quality of Life Scale (ProQol-V) were examined using the chi-square (χ^2^) test of homogeneity. The Blom’s method (Q–Q plot) was applied to assess the normality of scale scores, and Cronbach’s alpha coefficients were computed to evaluate internal consistency reliability. Despite the slight skewness that was observed in the data, parametric methods were implemented for comparisons and correlations among variables and participants’ characteristics. Analysis of Variance (ANOVA) was employed to compare the subscales of the ProQol measure. Additionally, Pearson’s correlation coefficient was used to examine univariate relationships between the ProQol subscales, as well as between these subscales and participants’ sociodemographic characteristics. Finally, multiple linear regression analyses were conducted to estimate standardized beta coefficients representing the associations between the ProQol subscales, participants’ characteristics, and the fear of coronavirus transmission. The level of statistical significance was set at *p* < 0.05.

## 3. Results

### 3.1. Demographic and Professional Characteristics of Health Professionals

A total of 246 HPs participated in this study. The majority were women (81.3%), and 33.8% were aged 50 years or older (Table 1). Most participants were married or cohabiting (61.4%), and 65.9% reported having children. Regarding educational attainment, 57.7% held an undergraduate degree, while 24.8% possessed a postgraduate qualification.

Analysis of professional categories showed that the majority (56.1%) were nursing staff with Technological Education, 8.5% held university-level nursing qualifications, and 15.4% were Nursing Assistants. Medical personnel accounted for 11.4%, while other professional groups—such as Social Workers, Health Visitors, and Technologists—represented 8.5%. In terms of employment status at the time of the study, 68.3% reported holding permanent positions, while 15.1% were auxiliary staff. The majority, or 36.6%, of the HPs worked in the primary medical sector, and an additional 9.3% in the secondary medical sector (total 35.9%). In addition 27.2% of those who participated in the study were from the surgical sector, and 10.6% were from the laboratories of the hospital. Regarding managerial responsibility, most participants (84.6%) reported not holding a supervisory position. Among those who did, 8.5% were heads of department, 4.1% were supervisors, 1.2% were sector coordinators, and 1.6% were directors. Additionally, the survey participants exhibit substantially greater experience in the health sector, with 56.1% reporting more than 15 years of employment in this field, compared with only 29.7% having equivalent tenure in their current department (marginal homogeneity test, *p* < 0.001).

### 3.2. Fear of Coronavirus Transmission

**Figure 1:**
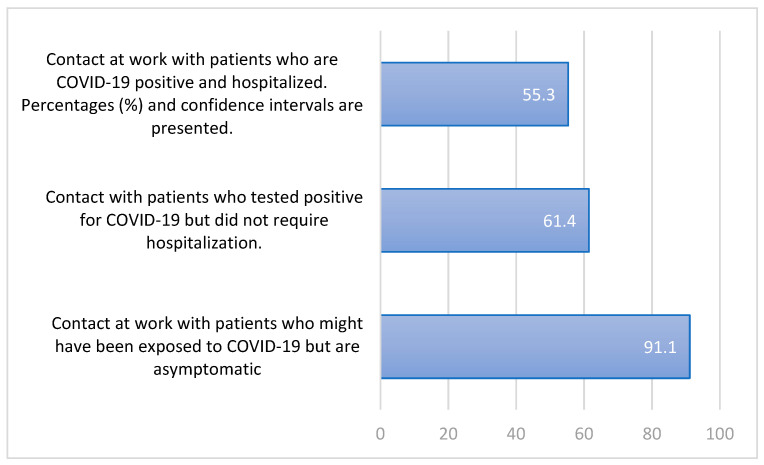
Distribution % of responses to questions related to the COVID-19 pandemic regarding contact with patients among the 246 healthcare professionals in the study.

A substantial proportion (91.1%) of HPs reported occupational contact with patients potentially exposed to COVID-19 but asymptomatic (Figure 1). This percentage was higher than those who reported contact with confirmed COVID-19 patients who were not hospitalized (61.4%) or with hospitalized COVID-19 patients (55.3%).

Analysis of the five items comprising the fear related to the COVID-19 pandemic (Table 2) revealed statistically significant variation across all items (*p* < 0.05). Specifically, 33.7% of participants reported “no concern” about the possibility of being quarantined, whereas 34.6% reported being “very concerned” about transmitting the virus to family or friends.

The cumulative score derived from the fear scale indicated a moderate level of fear, with a mean score of 14.8 (SD = 4.6), on a scale ranging from 5 (no fear) to 25 (very high fear). Reliability analysis using Cronbach’s alpha demonstrated excellent internal consistency (α = 0.826).

### 3.3. Professional Quality of Life

As with the fear scale, significant variation was observed across the 30 items (*p* < 0.05), ranging from “never” to “very often.” High frequencies of “often” or “very often” responses were found in items such as “I feel trapped” (23.5%), “I feel connected” (80.9%), and “I am a person who cares deeply” (80.3%) (Table 3).

CS showed moderate to high mean scores (M = 37.2, SD = 5.8), within a possible range of 10–50, where higher scores indicate greater satisfaction. *BO* presented moderate to low mean scores (M = 28.5, SD = 5.8), with higher scores denoting greater exhaustion. *STS* also reflected moderate to low mean scores (M = 24.5, SD = 6.4). Among the three subscales, CS exhibited significantly higher mean scores (*p* < 0.001). Reliability coefficients (Cronbach’s α) ranged from 0.769 to 0.850, indicating excellent internal consistency. Based on categorical classifications, 23.2% of participants demonstrated high CS, none exhibited high BO, and only 1.2% showed high STS (Table 4).

### 3.4. Relationship Between COVID-19-Related Fear and Professional Quality of Life

Concerning the relationship between fear derived from COVID-19 and ProQol (Table 5), holding a supervisory position was significantly associated with higher CS (*r* = 0.170, *p* < 0.05). BO was significantly correlated with having children (*r* = 0.171, *p* < 0.05), holding a permanent position (*r* = −0.184, *p* < 0.05), years of experience in the health sector (*r* = 0.164, *p* < 0.05), years of experience within the department (*r* = 0.153, *p* < 0.05), and higher COVID-19-related fear (*r* = 0.253, *p* < 0.05). Additionally, STS was positively correlated with age (*r* = 0.155, *p* < 0.05), years of professional experience (*r* = 0.167, *p* < 0.05), and increased levels of COVID-19-related fear (*r* = 0.281, *p* < 0.05).

Finally, multiple linear regression analyses (Table 6) identified predictors of the ProQol subscales. Higher CS was significantly associated with older age (*b* = 0.441, *p* = 0.004) and holding a supervisory position (*b* = −0.208, *p* = 0.003). Increased BO was predicted solely by higher levels of COVID-19-related fear (*b* = 0.259, *p* < 0.001), as was STS (*b* = 0.267, *p* < 0.001).

## 4. Discussion

The present study investigated the professional quality of life among HPs at a University General Hospital during the COVID-19 pandemic. A total of 246 HPs participated; most of them were women, and all possessed a high level of education. The majority of the study participants were nurses with university-level education who were working in the medical sector. At the time of the study, the participants had substantial experience in the healthcare sector, although they had comparatively fewer years of service within their current departments.

Overall, ProQol found to be moderate. CS showed moderate to high mean scores, BO presented moderate to low mean scores, and STS also reflected moderate to low mean scores. Among the three subscales, CS exhibited significantly higher mean scores. The specific characteristics of the sample (mostly female nurses working in the medical sector with a high level of education), may have influenced the results regarding the ProQol. However, these results are consistent with those reported in relevant studies [32].

In line with the results of the current study, numerous cross-sectional studies indicated that HPs typically experienced moderate CS alongside low to moderate levels of BO during the pandemic, implying that the intrinsic rewards of patient care may have alleviated stress for many HPs despite the pandemic’s pressures [33]. Systematic reviews of early pandemic research reveal that although BO and CF generally increased during high-stress periods, CS frequently remained stable or moderate, especially in the presence of resilience and supportive resources [34]. However, a higher level of CS has been associated with a stronger sense of meaningful contribution [35].

Similar results were reported in a study conducted among nurses at two Athens hospitals during the pandemic’s third wave, indicating moderate to high CS, moderate to low BO, and consistently low STS [32]. Accordingly, a study conducted in Greece, in the island of Crete, found moderate levels of CS, BO, and STS among 102 HPs in two Greek hospitals [36]. These findings are consistent with those reported in a relevant study, who surveyed 375 clinical nurses in Saudi Arabia and found average levels of CS, BO, and STS [37]. Comparable study evidence identified nurse-to-patient ratio as a significant predictor during the pandemic’s second wave [38]. In Spain, female HPs, reported higher STS than males, while frontline staff and nursing assistants demonstrated higher CS than second-line staff and physicians. BO and STS were also correlated with depression and/or anxiety [39]. A study examining emergency department staff in Hong Kong using the same research instrument as the current study found moderate levels of CS, BO, and STS [40].

In contrast, a multicenter study conducted in Spain involving 1521 nurses reported different results [41]. The ProQol was assessed, in conjunction with several socio-demographic and occupational characteristics. Elevated levels of CF and BO were observed, while CS levels remained below the estimated mean. Marital status, healthcare environment, geographical location of the center, and work shift were characteristics correlated with CF. Factors associated with CS included the professional’s age, gender, marital status, healthcare setting of the facility, geographical location of the center, and work shift. According to the exploratory model, employment in urban areas and work shift predicted reduced CS, while BO was influenced solely by work shift [41].

Heterogeneity of the results concerning ProQol during the COVID-19 pandemic period may also be explained by the timing of data collection. Studies conducted during peak periods—characterized by high service demand, inadequate personal protective equipment, and staff shortages—indicate elevated levels of BO and STS compared with research carried out during more stable times [34]. Furthermore, studies dominated by nurses—due to overrepresentation of women—may reveal different average levels compared to studies with mixed HPs samples [29]. Additionally, HPs working in Intensive Care Units, emergency departments, and COVID-19 wards consistently exhibit higher levels of burnout (BO) and secondary traumatic stress (STS) than non-frontline personnel, primarily due to increased workload and the demands associated with managing critically ill patients with coronavirus [42]. Moreover, researchers should not underestimate the impact that different interpretations of ProQol-related scales may have on prevalence estimates [34].

Regarding HPs’ characteristics, the present study revealed that holding a position of responsibility was significantly associated with higher CS, possibly due to the provision of greater autonomy, influence over work conditions, and a sense of meaningful contribution—factors known to enhance ProQol [35]. BO in the current study was related to having children, permanent employment status, more years of experience, and increased fear/anxiety of transmitting the coronavirus. These results align with the findings of a relevant study, in which having children and permanent employment status were associated with higher BO [34]. Consistent findings have been reported across various settings. At Tanta University Hospital, Egypt, years of experience was observed to be a key predictor of BO among physicians and nurses [43]. In an Iranian study, a significant association between professional tenure and BO among 1141 Iranian HPs [44] was demonstrated, while a U.S. study of 771 hospital staff across 47 North Dakota hospitals showed that years of experience significantly influenced CS, BO, and perceived stress, with CS lowest at 5–10 years of experience [45].

Concerning STS, the present research found that higher STS was associated with older age, longer work experience, and greater pandemic-related fear. In Lombardy, Italy, 39.8% of 653 HPs were diagnosed with STS. In contrast, regression analysis indicated higher levels of STS among women compared to men, and greater stress among frontline nurses than second-line nurses; consistent with the finding of the current study, a higher level of STS was also attributed to increased fear of infection [46].

Levels of COVID-19-related fear of infection were found to be moderate. These relatively moderate fear levels observed among HPs reflect the policy implemented during the data collection period, as only vaccinated HPs were permitted to work, and unvaccinated individuals were required to take mandatory leave. It is worth noting that in Greece, the period from March to June 2021 was defined by the country’s third wave of the pandemic. This wave was particularly significant because it brought the healthcare system under its greatest pressure yet, and led to the highest death toll seen in Greece up to that point. Nevertheless, during the third wave in Greece (March–June 2021), the fear of transmission among healthcare professionals (HCPs) was a complex phenomenon. While some studies suggest that fear of personal infection remained present, there were several factors that likely limited or mitigated that fear compared to the first and second waves; as HPs became more familiar with the virus’s behavior and the effectiveness of their safety protocols, the acute, paralyzing fear of transmission decreased [47]. Furthermore, in a related study, authors noted that by the third wave, high vaccination uptake (over 90% in some medical cohorts) significantly lowered the perceived personal threat [48].

In a similar cross-sectional study conducted in Denmark involving 4303 HPs [49], 49% reported fear of infection and 68% feared transmitting the virus to their families, particularly among ambulance workers. Fear of transmitting infection was also higher among elderly care staff. Self-reported exposure to infection and limited access to testing were associated with fear of both infection and transmission, while insufficient access to personal protective equipment (PPE) was linked exclusively to fear of transmission. Close contact with and exposure to COVID-19 patients led HPs to experience elevated levels of fear and anxiety, often resulting in psychological symptoms such as stress, panic, and anxiety disorders [50]. Additionally, excessive workload, shortage of PPE, exposure to negative media content regarding COVID-19, and insufficient psychological support have been identified as contributing factors to these symptoms [51]. Moreover, fear and anxiety were widespread among HPs, who are not immune to emotional distress and, in fact, may experience higher fear levels than the general population [52]. Fear is a natural response, while courage represents a deliberate choice—one that involves trusting evidence-based infection control practices to provide the highest possible standard of care during the pandemic. Consistent with the present results, a web-based study of 225 physicians from Pakistan, England, and the United States highlighted that the majority feared transmitting the virus to their family members [53]. It has also been reported that the perceived risk of infection to oneself and one’s family, combined with inadequate support from health authorities, substantially increased fear among HPs [30].

## 5. Limitations

This single-site cross-sectional study investigates Greek HPs at a critical period of the pandemic, using a well-recognized instrument (ProQol-V) and a brief module assessing COVID-related fear. Nonetheless, certain limitations must be acknowledged. The lack of pre-pandemic baseline data should be acknowledged, with precludes definite conclusions regarding the impact of COVID-19 on ProQol. Moreover, a single-site cross-sectional study does not allow for the establishment of causal relationships between the analyzed variables. Additionally, the use of a convenience modestly sized sample drawn from a single hospital, together with the underrepresentation of certain subgroups (e.g., males and other healthcare professional categories), limits the generalizability of the findings and restricts the stability of multivariable models. The reliance on self-reporting measures may also introduce potential bias due to subjective response tendencies.

Furthermore, the assessment of ProQol indicated that its variability depends on factors beyond those examined in the present study, as reflected by the low values of the coefficients of determination. Therefore, a re-evaluation and further investigation to identify additional explanatory factors of quality of life are considered necessary. Concerning the items assessing COVID-related fear, this questionnaire represents an exploratory measure rather than a validated scale. The findings related to these items should be interpreted with caution and viewed as indicative rather than definitive. Future studies should further evaluate the psychometric properties of this measure, including factor structure and construct validity, in independent samples.

Despite these limitations, the study provides valuable insights into the psychological impact of COVID-19 pandemic on the ProQol of HPs in a Greek hospital.

## 6. Conclusions

The present study examined the ProQol among HPs during the COVID-19 pandemic. Findings indicated moderate overall ProQol, with CS at moderate to high levels, BO at moderate to low levels, and STS at moderate to low levels. COVID-19-related fear was also moderate. Higher CS was associated with holding a position of responsibility. BO was associated with having children, permanent employment, years of experience, and increased pandemic-related fear. Higher STS was associated with older age, more years of experience, and greater pandemic-related fear or anxiety. These results highlight the need for immediate interventions by policymakers to improve HPs’ ProQol and reinforce healthcare system resilience.

Given that CS acts as a protective factor against BO and STS, healthcare policy should move beyond deficit-oriented strategies and proactively promote positive professional experiences. This may encompass institutional recognition initiatives, opportunities for career development, and recognition of the critical role of HPs, particularly during health crises.

The observed differences between younger and older professionals indicate a necessity for policies that provide structured mentorship, supervision, and resilience-building initiatives for less experienced personnel. Considering the correlation between leadership positions and increased CS, governments should prioritize investment in leadership training that emphasizes supportive management, emotional intelligence, and team well-being. Moreover, protecting leaders from excessive workload and role strain may help sustain their positive impact on team morale and ProQol. The relatively moderate levels of STS might indicate a strong sense of professional duty, yet dependence on duty alone is not sustainable. Policymakers must integrate accessible mental health services, peer-support systems, and designated recovery time into pandemic preparedness and response strategies.

The moderate levels of COVID-19-related fear highlight the necessity of regular psychological risk assessments during public health crises. Policies should involve regular assessments for BO, anxiety, and STS, facilitating early detection and prompt intervention.

Policies that retain and value experienced healthcare specialists—such as through incentives, flexible work arrangements, or advisory roles—may enhance institutional resilience in future crises by leveraging the protective advantages of experience and CS. These policy implications demand a transition towards proactive, experience-informed, and psychologically sustainable healthcare systems, where the well-being of HPs is considered as an essential element of emergency preparedness rather than a secondary concern. Finally, longitudinal or experimental studies are required to comprehensively address the study topics, as they would be able to provide more robust causal evidence and deep insight into the evolving impacts on ProQol over time.

## Figures and Tables

**Table 1:** General characteristics of 246 participating health professionals from a regional University General Hospital.

			*n*	%
Gender		Males/Females	46/200	18.7/81.3
Age, years		22–24	6	2.4
		25–29	36	14.6
		30–34	25	10.2
		35–39	23	9.3
		40–44	29	11.8
		45–49	44	17.9
		50–54	48	19.6
		55–59	31	12.6
		60+	4	1.6
Marital Status		Single, Divorced, Widowed	95	38.6
		Married or in cohabitation	151	61.4
Children		Yes	162	65.9
		No	84	34.1
Education	Institute of vocational training, Hospital nursing school		43	17.5
		Tech. University,	112	45.5
		University	30	12.2
		MSc	48	19.5
		PhD	13	5.3

**Table 2:** Distribution of responses to the five items of the fear scale related to the COVID-19 pandemic among 246 health professionals.

Statements	Not at All	A Little	Fairly	Much	Very Much
4.1 I am afraid that I may be infected with the coronavirus.	15.4%	40.7%	25.6%	12.2%	6.1%
4.2 I am afraid that I may transmit coronavirus to my family/friends.	4.9%	16.3%	26.0%	18.3%	34.6%
4.3 I am afraid that I may be quarantined	33.7%	29.7%	18.7%	9.8%	8.1%
4.4 I am afraid that if I become infected, I might infect my colleagues	8.5%	26.8%	29.7%	22.8%	12.2%
4.5 I am afraid that if I become infected, I might infect my patients	11.4%	15.4%	24.8%	25.6%	22.8%

**Table 3:** Distribution of responses to the Professional Quality of Life Scale (ProQol) items.

Statements:	Never	Rarely	Sometimes	Often	Very Often
1. I am happy *	1.6%	7.7%	32.1%	45.9%	12.6%
2. I worry a lot	1.6%	13.4%	33.7%	36.2%	15.0%
3. I feel connected	0.4%	2.8%	15.9%	45.5%	35.4%
4. I get satisfaction *	3.3%	13.0%	33.3%	33.7%	16.7%
5. I startle easily	13.8%	34.1%	32.1%	11.8%	8.1%
6. I feel refreshed	2.4%	8.5%	35.8%	39.8%	13.4%
7. I find it hard to separate (work/life)	29.7%	37.0%	19.1%	11.8%	2.4%
8. I am not as productive	38.2%	41.5%	14.2%	3.3%	2.8%
9. I think I have been affected	29.3%	38.6%	24.4%	6.5%	1.2%
10. I feel trapped	24.0%	25.2%	27.2%	15.4%	8.1%
11. I have felt irritable	10.6%	23.2%	36.6%	22.8%	6.9%
12. I like my job	1.2%	4.1%	11.4%	42.3%	41.1%
13. I feel depressed	17.9%	32.9%	34.1%	12.2%	2.8%
14. I feel as though I am experiencing a trauma	24.4%	41.9%	23.2%	7.3%	3.3%
15. I have strong beliefs *	1.2%	6.5%	29.3%	45.9%	17.1%
16. I am satisfied	2.4%	5.3%	30.5%	49.2%	12.6%
17. I am the person I always wanted to be *	2.0%	7.7%	26.0%	45.1%	19.1%
18. My work makes me feel satisfied	2.4%	4.5%	30.1%	44.3%	18.7%
19. I feel exhausted	3.7%	14.2%	32.5%	30.9%	18.7%
20. I have pleasant thoughts	0.8%	4.1%	32.1%	48.8%	14.2%
21. I feel worn out	4.9%	19.9%	34.1%	21.5%	19.5%
22. I believe I can make a difference	2.0%	14.2%	40.2%	35.4%	8.1%
23. I avoid certain activities	30.9%	41.1%	17.9%	8.5%	1.6%
24. I am proud	0.4%	6.1%	22.0%	43.1%	28.5%
25. I have disturbing, frightening thoughts	41.5%	33.3%	16.3%	8.1%	0.8%
26. I feel stuck	9.3%	24.4%	26.4%	23.6%	16.3%
27. I have thoughts that I am successful	3.7%	9.8%	42.3%	36.6%	7.7%
28. I cannot recall (certain experiences)	20.7%	36.2%	30.1%	11.0%	2.0%
29. I am a person who cares deeply *	0.8%	3.3%	15.9%	42.3%	37.8%
30. I am happy with my career choice	4.9%	8.1%	25.2%	35.0%	26.8%

* For the scoring of the ProQol, the responses to these specific items are reverse-coded.

**Table 4:** Mean scores of the ProQol subscales among 246 health professionals.

	Mean	SD	Median	Min.	Max.	Cronbach α
Compassion Satisfaction	37.2	5.8	38.0	16	50	0.850
Low (up to 22)	*n* = 3 or 1.2%					
Moderate (23–41)	*n* = 186 or 75.6%					
High (42+)	*n* = 57 or 23.2%					
Burnout Syndrome	28.6	4.5	28.5	18	40	0.769
Low (up to 22)	*n* = 24 or 9.8%					
Moderate (23–41)	*n* = 222 or 90.2%					
High (42+)	--					
Secondary Traumatic Stress	24.5	6.4	24.0	10	45	0.828
Low (up to 22)	*n* = 99 or 40.3%					
Moderate (23–41)	*n* = 144 or 58.5%					
High (42+)	*n* = 3 or 1.2%					

Higher scores indicate greater symptomatology. Analysis of Variance among the three subscales, *p* < 0.001.

**Table 5:** Bivariate correlations between demographic/professional characteristics, COVID-19-related fear, and ProQol scores.

	Compassion Satisfaction	Burnout Syndrome	Secondary Traumatic Stress	Fear About COVID-19
	r-Pearson			
GENDER (1: males, 2: females)	−0.009	0.089	0.041	−0.012
Age (per five years intervals)	0.052	0.118	0.155 *	0.031
Marital status (1: Single, Divorced, Widowed, 2: Married or in cohabitation)	−0.012	0.116	0.115	0.071
Children (1: yes, 0: no)	0.005	0.171 *	0.113	0.047
Education (1: Institute of vocational training, Hospital nursing; 2: Tech. Univ.; 3: Univ.; 4: MSc; 5: PhD)	−0.071	−0.051	−0.050	−0.060
Employment Status (1: permanent, 2: non-permanent, etc.)	0.078	−0.184 *	−0.092	0.036
Position of responsibility (1: yes, 0: no)	0.170 *	−0.021	0.046	−0.022
Years in Health sector (categorized as “up to 1”, 2–3, 4–5, 6–10, 11–15, 16–20, 21–25, 26–30 and >30)	−0.025	0.164 *	0.167 *	0.045
Years in the department (categorized as “up to 1”, 2–3, 4–5, 6–10, 11–15, 16–20, 21–25, 26–30 and >30)	−0.074	0.153 *	0.090	−0.010
Fear about COVID-19	−0.057	0.253 *	0.281 *	--

Higher scores indicate greater symptomatology. * *p* < 0.05.

**Table 6:** Multiple linear regression analysis of the ProQol subscales in relation to health professionals’ characteristics and COVID-19-related fear.

	Compassion Satisfaction		Burnout Syndrome		Secondary Traumatic Stress	
Predictive Factors	beta	*p*-value	beta	*p*-value	beta	*p*-value
Gender (1: males, 2: females)	−0.034	0.598	0.091	0.148	0.043	0.499
Age (per five years intervals)	0.441	0.004	−0.222	0.134	0.059	0.696
Marital Status (1: Single, Divorced, Widowed, 2: Married or in cohabitation)	−0.030	0.768	−0.067	0.501	0.062	0.536
Children (1: yes, 2: no)	−0.021	0.850	−0.152	0.163	0.019	0.862
Education (1: Institute of vocational training, Hospital nursing 2: Tech. Univ., 3: Univ., 4: MSc, 5: PhD)	−0.101	0.131	0.012	0.848	0.004	0.949
Employment Status (1: permanent, 2: non-permanent, etc.)	0.181	0.073	−0.166	0.095	0.052	0.606
Position of responsibility (1: yes, 2: no)	−0.208	0.003	0.087	0.202	0.012	0.857
Years in Health sector (categorized as “up to 1”, 2–3, 4–5, 6–10, 11–15, 16–20, 21–25, 26–30 and >30)	−0.277	0.078	0.126	0.410	0.159	0.307
Years in the department (categorized as “up to 1”, 2–3, 4–5, 6–10, 11–15, 16–20, 21–25, 26–30 and >30)	−0.131	0.153	0.112	0.212	−0.035	0.704
Fear about COVID-19 (Higher scores indicate higher Fear)	−0.067	0.284	0.259	<0.001	0.267	<0.001
R^2^(R^2^ adjusted)	0.101 (0.063)		0.135 (0.098)		0.109 (0.071)	

ProQol Scale score from 1: Never to 5: Very often. Higher score → higher symptomatology.

## Data Availability

The data presented in this study are available on request from the corresponding author.
